# Antidiabetic Treatment and Prevention of Ischemic Stroke: A Systematic Review

**DOI:** 10.3390/jcm13195786

**Published:** 2024-09-28

**Authors:** Vasiliki Prentza, George Pavlidis, Ignatios Ikonomidis, Sotirios Pililis, Stamatios Lampsas, Aikaterini Kountouri, Loukia Pliouta, Emmanouil Korakas, John Thymis, Lina Palaiodimou, Aikaterini Tsegka, Konstantinos Markakis, Panagiotis Halvatsiotis, Georgios Tsivgoulis, Vaia Lambadiari

**Affiliations:** 1 2nd Department of Cardiology, Attikon University Hospital, Medical School, National and Kapodistrian University of Athens, 12462 Athens, Greece; prentzavasiliki@gmail.com (V.P.); ignoik@gmail.com (I.I.); lampsas.stam@gmail.com (S.L.); johnythg@gmail.com (J.T.); 2 Research Unit and Diabetes Center, 2nd Department of Internal Medicine, Attikon University Hospital, Medical School, National and Kapodistrian University of Athens, 12462 Athens, Greece; sotiris181@yahoo.gr (S.P.); katerinak90@hotmail.com (A.K.); plioutaloukia@gmail.com (L.P.); mankor-th@hotmail.com (E.K.); tsegka@gmail.com (A.T.); kmark2105@gmail.com (K.M.); pahalv@gmail.com (P.H.); vlambad@otenet.gr (V.L.); 3 2nd Department of Neurology, Attikon University Hospital, Medical School, National and Kapodistrian University of Athens, 12462 Athens, Greecetsivgoulisgiorg@yahoo.gr (G.T.)

**Keywords:** antidiabetic drugs, diabetes, ischemic stroke, prevention, metformin, pioglitazone, dipeptidyl peptidase 4 inhibitors, glucagon-like peptide-1 receptor agonists, sodium-glucose co-transporter 2 inhibitors, tirzepatide

## Abstract

**Background:** Diabetes mellitus (DM) is a prevalent disease in the general population and also a well-established risk factor for the development of ischemic stroke. Patients who have been diagnosed with diabetes have a 20% higher risk for developing ischemic stroke in comparison to non-diabetic individuals. The aim of the current systematic review is to provide the latest evidence regarding the association between antidiabetic treatment and the prevention of ischemic stroke. **Methods:** A comprehensive search in scientific literature databases PUBMED, COCHRANE, and SCOPUS was conducted. The studies that were deemed as eligible for this review were those that examined the clinical benefits of therapeutic strategies in terms of preventing ischemic strokes. **Results:** A total of 32 studies met the established selection criteria. The included studies showed that pioglitazone treatment significantly reduced the risk for recurrent stroke in patients with DM. Furthermore, in the context of primary prevention, the improvement in glycemic control after treatment with the glucagon-like peptide-1 receptor agonists (GLP-1RA) semaglutide and dulaglutide was associated with a reduction in the risk of ischemic stroke in diabetic subjects. Metformin monotherapy may reduce stroke risk, while dipeptidyl peptidase 4 inhibitors, sodium-glucose co-transporter 2 inhibitors, and insulin do not seem to affect the incidence of stroke. **Conclusions:** The findings of the present systematic review suggest that pioglitazone and GLP-1RA may decrease the risk of stroke. Further studies are needed to provide additional data regarding the preventive effect of novel antidiabetic drugs, such as dual glucose-dependent insulinotropic polypeptide/GLP-1RA agents, on stroke.

## 1. Introduction

Diabetes mellitus (DM) is a common disease that has shown an increased prevalence in the last decades. The increased frequency of DM is caused mainly by the aging of the general population, as well as by the elevated frequency of obesity. According to the International Diabetes Federation, the prevalence of DM in the adult population was estimated to be 10.5% in 2021, rising to 12.2% in 2045, while the total number of people who suffer from the disease on a global scale has been predicted through mathematical models to increase from 463 million in 2019 to 578 million by 2030 and is estimated to reach 783 million by 2045 [1].

Diabetes mellitus is a well-established independent risk factor for the development of acute cerebrovascular events, such as ischemic stroke. Notably, in comparison to non-diabetic individuals, patients with DM present a twofold increase in their risk of developing a type of stroke, which accounts for approximately 20% of deaths in those patients. Hence, stroke is one of the prominent factors of mortality in patients of this category. The duration of diabetes is also addressed as a risk factor for the development of ischemic stroke. In particular, every year of diabetes duration may increase the risk of ischemic stroke by 3%. Furthermore, hyperglycemia has been proposed to increase the severity of ischemic stroke and the clinical outcomes that follow a stroke [2,3].

Several clinical trials in the last decade have suggested that tight glycemic control, and more specifically, glycated hemoglobin (HbA1c) levels < 6–6.5%, in comparison to HbA1c < 7–8%, are associated with a significant decrease in the risk for damage at a microvascular level and a further reduction in non-fatal coronary events, such as myocardial infarction. However, tight glycemic control does not decrease the mortality rate but increases the risk of patients to develop symptomatic hypoglycemia. These observations are also evident in the recommendations of several diabetes associations, which suggest that for the majority of patients with DM, a target value of HbA1c < 7% is considered reasonable in order to significantly reduce the risk of developing of cardiovascular (CV) events [4]. 

**Figure 1:**
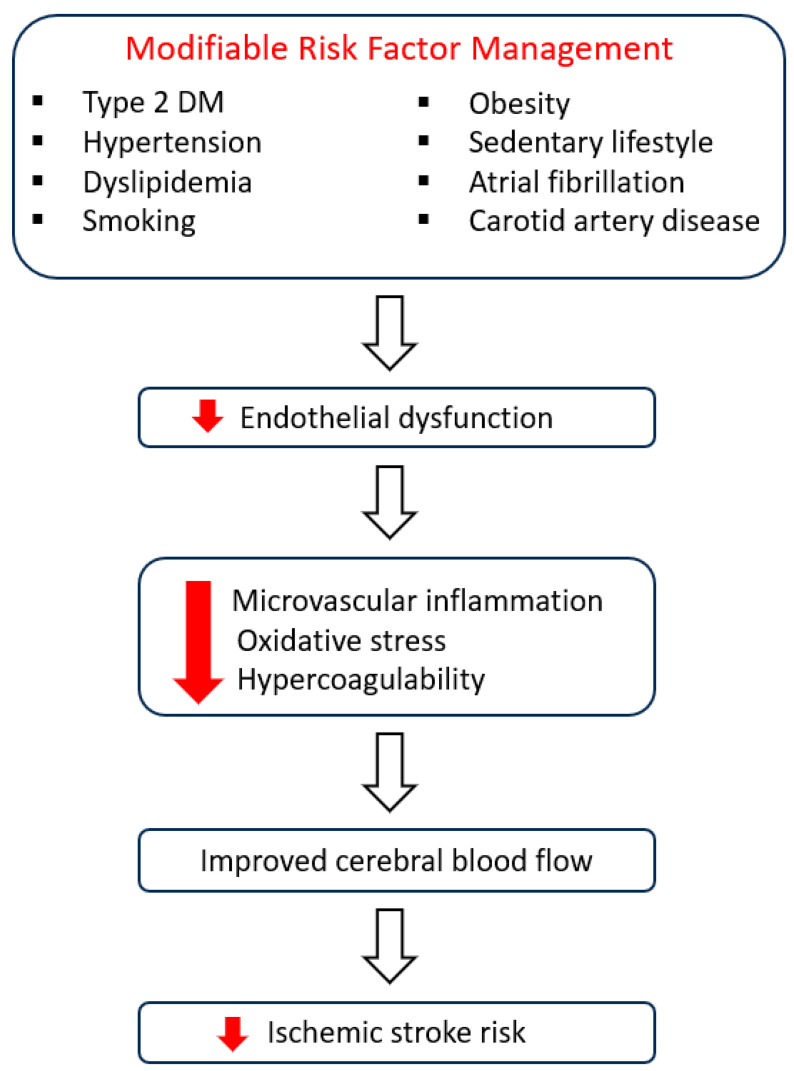
Modifiable risk factor management confers a significant improvement of endothelial function, leading to reduced ischemic stroke risk. DM, diabetes mellitus.

It is also evident that the management of stroke and DM share many characteristics due to the fact that both diseases affect the conditions of the blood vessels. Moreover, DM is commonly associated with an increased prevalence of other co-existing CV risk factors, such as dyslipidemia, hypertension, and obesity [5]. Cardiovascular diseases and ischemic stroke in the general population have an increased mortality rate, and for this reason, the optimization and aggressive management of their risk factors are of great importance for the health of the general population. As a matter of fact, it has been shown that smoking, increased age, elevated systolic blood pressure, and atrial fibrillation (AF) are determined as major risk factors for the development of a first ischemic stroke. With the prominent exception of age, all risk factors are modifiable and may be used as factors for the development of a routine protocol for stroke prevention in non-diabetic patients [6,7]. Indeed, appropriate lifestyle modifications and risk factor management improve endothelial function, which in turn reduces microvascular inflammation, oxidative stress, and hypercoagulability and improves cerebral blood flow, leading to reduced ischemic stroke risk (Figure 1) [8].

Accumulating evidence shows that the newer glucose-lowering agents, namely dipeptidyl peptidase-4 inhibitors (DPP-4i), glucagon-like peptide-1 receptor agonists (GLP-1RA), and sodium-glucose cotransporter-2 inhibitors (SGLT-2i), have potential anti-inflammatory properties linked to intrinsic actions of the pharmacological class beyond glycemic control [9]. In particular, treatment with GLP-1RA and SGLT-2i confers a significant improvement in endothelial function and arterial stiffness due to their direct and indirect vascular effects. Interestingly, the combination of GLP-1RA and SGLT-2i seems to be superior and additive to the separate administration of each drug, and the beneficial effects on endothelial function and oxidative stress appear earlier [10,11].

The aim of the present systematic review was to search the current literature in order to provide insights into the relationship between antidiabetic treatment and the prevention of ischemic stroke.

## 2. Methods

The present systematic review was conducted in accordance with the PRISMA (Preferred Reporting Items for Systematic Reviews and Meta-Analyses) criteria. This review was not registered. Institutional Review Board approval was waived owing to the study design (systematic review). 

For the purpose of this review, two independent reviewers (V.P. and G.P.) conducted a comprehensive search of the literature in the scientific publication databases PUBMED, COCHRANE, and SCOPUS using the following algorithm: “diabetes” OR “blood glucose control” OR “glycated hemoglobin” OR “Antidiabetic Drug” OR “Insulin Resistance” AND “Ischemic Stroke” OR “Risk factors for ischemic stroke” OR “Prevention”. The Boolean operator OR was used to refine the results with similar terms (similar meaning), and the Boolean operator AND was used to refine the results with one or more sequence of terms that included completely different meanings but were a part of the research question. The search extended from the inception of each electronic database to 31 December 2023.

The studies that were deemed as eligible for this review were those that examined the clinical benefits of therapeutic strategies in terms of preventing ischemic strokes. For the determination of the included studies, the PICO (population, intervention, comparison, and outcome) approach was used and more specifically, the studies that were included concerned adult patients (P) of both sexes with DM. Regarding the intervention (I) and control (C) factors, the studies that were included proposed interventions that aimed at identifying or decreasing the risk factors associated with DM and ischemic stroke. 

The exclusion criteria were studies with a publication date earlier than 1 January 2004 and studies written in a language other than English. Also, studies that were characterized as reviews, editorials, research protocols or commentary studies, non-reviewed studies, and conference abstracts were also excluded. The fields of extracted data included authors, year of publication, gender, age, sample size, and outcome measures. The study protocol was a priori designed, and all supporting data are available within the article.

The present study was thoroughly evaluated for potential sources of bias that could impact the validity of its results. Two independent reviewers (V.P. and G.P.) examined the included studies, with each study being reviewed by a different subject. Any discrepancies in the findings were discussed and resolved by senior reviewers (I.I. and V.L.). No specific tools were used to assess the methodological quality, including the risk of bias of the studies.

## 3. Results

**Figure 2:**
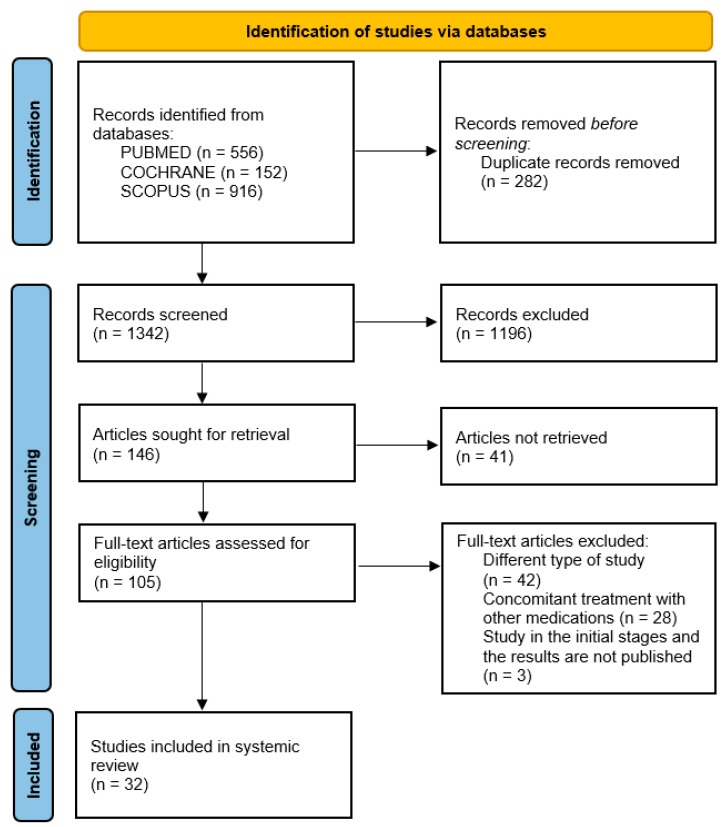
PRISMA flow diagram presenting the selection of eligible studies.

The initial database search identified 1624 records (556 from PUBMED, 152 from COCHRANE, and 916 from SCOPUS), which decreased to 1342 after the exclusion of duplicates. During the screening of the title and abstract, an additional 1196 irrelevant articles were excluded, while 41 papers could not be retrieved. After a thorough reading of the full texts of the remaining 105 articles, 73 did not meet the eligibility criteria and were consequently excluded. The remaining 32 studies fulfilled the eligibility criteria and were included in this systematic review, as shown in Figure 2. Regarding the type of included studies, 27 were randomized clinical trials (RCTs) [12,13,14,15,16,17,18,19,20,21,22,23,24,25,26,27,28,29,30,31,32,33,34,35,36,37,38], 2 were post hoc analyses of RCTs [39,40], and 3 were observational studies [41,42,43]. 

### 3.1. Pathological Effects of Hyperglycemia in the Cardiovascular System

In order to explain the negative effects of the elevated blood glucose levels in the acceleration of atherosclerosis, multiple pathways (indirect and direct) have been proposed. The pathways characterized as indirect include the development of atherogenic dyslipidemia, where there is evidence of a reduction in high-density lipoproteins, the formation of small low-density lipoproteins, and an increase in the levels of triglycerides. Furthermore, increased glucose levels are also linked to a dysfunction of the sympathetic nervous system. The acceleration of the atherosclerotic process can also occur through direct pathways, such as endothelial dysfunction, which consequently promotes prothrombotic, pro-inflammatory, and vasoconstrictive processes, contributing to the formation and eventually rupture of atheromatic plaques [44]. In addition, insulin resistance is associated with impaired endothelial glycocalyx, which leads to the deterioration of endothelial function and eventually the occurrence of CV disease [45]. Interestingly, patients with embolic stroke of an undetermined source present endothelial glycocalyx damage, compared to matched healthy controls with similar atherosclerotic risk factors [46].

### 3.2. Prevention of Ischemic Stroke in Diabetic Patients

#### 3.2.1. Glycemic Control

The optimization of glycemic control has been shown to reduce the risk for developing complications that are common to diabetic patients, including nephropathy, retinopathy, and neuropathy. However, there has been no recent study to show that an improved glycemic control has the capability to reduce the incidence of acute stroke or to improve the survival of acute stroke. Two studies have failed to demonstrate any positive correlation between improved glycemic control and decrease in the risk of ischemic stroke [12,39]. In the case of acute stroke, the aggressive management of glucose levels has also not shown any benefits. In the SHINE (Stroke Hyperglycemia Insulin Network Effort) trial, the patients were randomly selected to follow the therapeutic modalities of continuous intravenous insulin (intensive glucose control) or subcutaneous insulin on a sliding scale (standard glucose control) for up to 72 h. The trial failed to present any statistically significant difference in functional outcomes between the two therapeutic approaches in the time frame of 90 days [12]. 

The reasons that are suggested by the aforementioned studies in relation to the failure to correlate glycemic control with the outcomes of ischemic stroke are based on the fact that tight glucose control is complex, and its mechanisms not fully understood. According to the proposed hypothesis, hypoglycemia is a major pathogenetic factor that is more frequent in cases where tight glucose control is applied to diabetic patients. Moreover, hypoglycemia is not directly correlated to stroke. Another hypothesis is the fact that drugs that are commonly used to treat diabetes may be harmful themselves. A prominent example includes fluid retention caused by antidiabetic drugs that may increase the possibilities of heart failure or increase body weight associated with insulin therapy, which can be linked to a higher risk for ischemic stroke [12,39].

#### 3.2.2. Hypertension

Hypertensive management has been shown to reduce the risk of ischemic stroke in diabetic patients, as shown in several studies. These studies demonstrated that hypertension management with an angiotensin-converting enzyme inhibitor reduced the risk for the development of ischemic stroke by 33% [13,41]. However, a debate is evident in the scientific community regarding the optimum levels of blood pressure in patients with DM. Until recently, the target was as low as 130/80 mmHg; however, the trials included in this review suggested that there was no benefit of lowering the blood pressure below 120–140 mmHg in elderly patients. Even though the guidelines regarding the treatment of hypertension differ across organizations and countries, the basic principle is to address hypertension aggressively using medication that is appropriate to each patient individually. On the other hand, it has been shown that beta blocker medications are related to an increased risk of hypoglycemia and, therefore, are to be avoided. In cases where diabetic patients have macroalbuminuria, microalbuminuria, or renal disease, the first line of treatment that should be used is inhibition of the renin–angiotensin–aldosterone system [13,41].

### 3.3. Antidiabetic Drugs for Stroke Prevention

#### 3.3.1. Metformin

In the UKPDS (United Kingdom Prospective Diabetes Study), the administration of metformin to overweight patients with type 2 DM reduced the risk of stroke compared to treatment with sulphonylureas or insulin (*p* = 0.032) [47]. On the contrary, the more recently published Cochrane review suggested no clear evidence regarding monotherapy with metformin and patient-specific outcomes, like non-fatal stroke [48]. Nevertheless, in a meta-analysis of RCTs and observational studies, metformin appears to reduce the risk of stroke in the context of primary prevention. More specifically, metformin monotherapy was effective in decreasing stroke risk in both RCTs (relative risk (RR) = 0.66; 95% confidence interval (CI), 0.50–0.87; *p* = 0.004) and cohort studies (RR = 0.67; 95% CI, 0.55–0.81; *p* < 0.0001). However, the favorable effect of metformin in stroke prevention was not observed in patients who received a combined treatment with metformin and other antihyperglycemic agents [49].

#### 3.3.2. Thiazolidinediones

Pioglitazone is a peroxisome proliferator-activated receptor gamma (PPARγ) antagonist that exhibits multiple effects and could play a role in the reduction of CV events and in the decrease of inflammatory markers levels, such as high-sensitivity C-reactive protein (CRP). Additionally, pioglitazone is an insulin-sensitizing agent mainly used for the regulation of blood glucose levels. 

In the PROactive study (Prospective Pioglitazone Clinical Trial in Macrovascular Events), 5238 patients with type 2 DM who had a high risk of macrovascular events were randomized to receive pioglitazone or placebo for a period of 34.5 months. The main secondary endpoint (all-cause mortality, non-fatal myocardial infarction, and stroke) presented at a lower rate in the pioglitazone group (HR, 0.84; 95% CI, 0.72–0.98; *p* = 0.027). Regarding the occurrence of stroke, in the pioglitazone group, it was reported in 76/2605 (2.9%) patients, while in the placebo group, it was reported in 96/2633 (3.6%) patients, which indicates a reduction of 20%, but the difference was not statistically significant [14]. According to a post hoc analysis of PROactive data, in patients with a prior stroke, pioglitazone was associated with a decrease in fatal and non-fatal stroke (HR, 0.53; 95% CI, 0.34–0.85; *p* = 0.0085). However, in patients without a previous stroke, no treatment effect was observed on the occurrence of a first stroke [40].

The J-SPIRIT (Juntendo Stroke Prevention Study in Insulin Resistance and Impaired Glucose Tolerance) randomized controlled trial included 120 patients with diagnosed impaired glucose tolerance or recently diagnosed type 2 DM and a history of ischemic or transient stroke. The patients received pioglitazone treatment for a duration of 2.8 years and the authors concluded that there was a decrease in the recurrent stroke rates (HR, 0.62; 95% CI, 0.13–2.35). However, this difference was statistically non-significant (*p* = 0.42) [15]. 

The IRIS (Insulin Resistance Intervention After Stroke trial) was a randomized clinical trial performed in non-diabetic patients with insulin resistance and a history of recent ischemic stroke within the previous 6 months. As patients with insulin resistance are considered to have high CV risk, pioglitazone not only improved glycemic control but also reduced insulin resistance. The IRIS study revealed that treatment with pioglitazone may reduce the risk of acute coronary artery disease and ischemic stroke in patients with insulin resistance and a recent stroke [16]. In the study, the primary endpoints related to fatal or non-fatal stroke or myocardial infarction occurred in 9% of the pioglitazone group and 11.8% of the placebo group during the 4.8 years that the study lasted (HR, 0.76; 95% CI, 0.62–0.93; *p* = 0.007). However, the rate of stroke alone did not differ between the pioglitazone and placebo groups (HR, 0.82; 95% CI, 0.61–1.10; *p* = 0.19).

A meta-analysis of three clinical trials showed that treatment with pioglitazone in a group of 4980 patients with a history of previous stroke and diagnosed insulin resistance was associated with a lower risk of recurrent stroke by 32% (HR, 0.68; 95% CI, 0.50–0.92; *p* = 0.01) and a reduced risk of major vascular events by 25% (HR, 0.75; 95% CI, 0.64–0.87; *p* = 0.0001). In the concluding remarks of this particular study, the authors mentioned that pioglitazone treatment, when used as an option, may reduce the risk of recurrent stroke and also may decrease the risk for major vascular events in patients with previous ischemic stroke and insulin resistance. Nonetheless, no similar evidence was mentioned in regard to the effects on all-cause mortality and heart failure [50]. 

Several mechanisms have been proposed for the positive effect of pioglitazone treatment in the prevention of ischemic stroke in subjects with insulin resistance. The most prominent one is based on the fact that pioglitazone is a pharmaceutical agent that improves insulin sensitivity, increases the concentrations of high-density lipoproteins, and decreases the concentrations of CRP and triglycerides. Furthermore, pioglitazone has been shown to reduce the risk of developing pre-diabetes compared to placebo [14,16,50].

#### 3.3.3. Dipeptidyl Peptidase 4 Inhibitors

Cardiovascular outcomes trials regarding DPP-4i have not shown CV benefits compared to placebo. The primary composite endpoint was CV death, non-fatal myocardial infarction, and non-fatal stroke, while the effects of therapeutic interventions on the occurrence of stroke alone were included in the secondary endpoints. More specifically, in the SAVOR-TIMI 53 (Saxagliptin Assessment of Vascular Outcomes Recorded in Patients With Diabetes Mellitus—Thrombolysis in Myocardial Infarction) trial, the safety of CV and efficacy of saxagliptin were evaluated. Saxagliptin did not change the rate of ischemic events, although the rate of hospitalization for heart failure was elevated [17]. The EXAMINE (Examination of Cardiovascular Outcomes with Alogliptin versus Standard of Care) trial showed non-inferiority of the DPP-4 inhibitor alogliptin to placebo in major adverse cardiac event (MACE) rates in patients with type 2 DM and recent acute coronary syndromes (HR, 0.96; upper boundary of the one-sided repeated CI, 1.16; *p* < 0.001 for non-inferiority and *p* = 0.32 for superiority) [18]. In the TECOS (Trial Evaluating Cardiovascular Outcomes with Sitagliptin) randomized, double-blind study, a total number of 14,671 patients were assigned to add either sitagliptin or placebo to their existing therapy. The primary CV outcome was a composite of CV death, non-fatal myocardial infarction, non-fatal stroke, or hospitalization for unstable angina. Among the patients with type 2 DM and established CV disease, adding sitagliptin to their usual care did not appear to increase their risk of MACE, hospitalization for heart failure, or other adverse events [19]. 

More recently, the CARMELINA (Cardiovascular and Renal Microvascular Outcome Study With Linagliptin) study revealed that among subjects with type 2 DM and high CV and renal risk, linagliptin added to the standard care, compared to placebo, resulted in a non-inferior risk of a composite CV outcome over a median follow-up period of 2.2 years [20]. In addition, the CAROLINA (Cardiovascular Outcome Study of Linagliptin Versus Glimepiride in Type 2 Diabetes) study demonstrated that the use of linagliptin, compared to glimepiride, over a median of 6.3 years led to a non-inferior risk of a composite CV outcome [21]. In conclusion, DPP-4i can be safely administered to stroke patients without providing any benefit beyond glycemic control.

#### 3.3.4. Glucagon-like Peptide-1 Receptor Agonists 

In the ELIXA (Evaluation of Lixisenatide in Acute Coronary Syndrome) study, lixisenatide treatment showed no improvement in the rate of primary CV outcomes or in the prevention of stroke [22]. Furthermore, in the LEADER (Liraglutide Effect and Action in Diabetes) study, no significant reduction in the risk for the development of ischemic stroke was observed in patients who were treated with liraglutide in comparison to patients who received placebo [23]. 

The SUSTAIN-6 (Semaglutide Unabated Sustainability in Treatment of Type 2 Diabetes) study evaluated the CV effects of semaglutide. A total of 3297 patients with high CV risk received semaglutide (0.5 mg or 1.0 mg) or placebo once weekly for 104 weeks. Semaglutide reduced the risk of the primary endpoint (CV death, non-fatal myocardial infraction, or stroke) by 26% compared to placebo. In addition, the risk of ischemic stroke was reduced by 39% in patients receiving semaglutide (HR, 0.61; 95% CI, 0.38–0.99; *p* = 0.04) [24]. Although the study presented significant beneficial effects on non-fatal stroke, the CV outcome trials were underpowered for the specific stroke endpoints. On the other hand, in the SELECT (Semaglutide Effects on Heart Disease and Stroke in Patients with Overweight or Obesity) trial, weekly subcutaneous semaglutide administration at a dose of 2.4 mg in overweight or obese patients with preexisting CV disease but without diabetes reduced the risk of death from CV causes, non-fatal myocardial infarction, or non-fatal stroke by 20% compared to placebo in a mean follow-up period of 39.8 months. However, the reduction of non-fatal stroke was not statistically significant over the length of the trial [25].

The EXSCEL (Exenatide Study of Cardiovascular Event Lowering) trial showed no statistically significant differences in the incidence of non-fatal ischemic stroke between patients with type 2 DM who were treated with exenatide via subcutaneous injection once per week and those who received placebo [26].

In the REWIND (Cardiovascular Research Events with Weekly Incretin in Diabetes) study, a total number of 9901 patients were enrolled. The patients had a recent diagnosis of type 2 DM and either a previous CV event or CV risk factors. There were treated with dulaglutide (single weekly dose of 1.5 mg) or placebo. The results showed that treatment with dulaglutide showed a significant reduction of 26% in the incidence of myocardial infraction, non-fatal stroke, or death from CV disease [27]. An exploratory analysis of the REWIND trial suggested that long-term use of dulaglutide may reduce the clinically relevant risk of ischemic stroke in patients with type 2 DM, while the severity of stroke was not affected [28]. 

The PIONEER-6 (Peptide Innovation for Early Diabetes Treatment) study was a multinational, randomized, controlled, double-blind trial in patients with type 2 DM with a high risk of CV events (age ≥ 50 years with established CV disease or moderate chronic kidney disease, or age > 60 years with ≥1 other CV risk factor). The patients were randomized to receive oral semaglutide (up to 14 mg) once daily or placebo as an initial treatment. The primary composite endpoint was the time to occurrence of the three components of the primary outcome (CV death, non-fatal myocardial infraction, or non-fatal stroke). The present trial demonstrated non-inferiority of oral semilattice to placebo with a hazard ratio (HR) 0.79 (95% CI, 0.57–1.11; *p* < 0.001) [29].

A recent study that analyzed previous reviews and meta-analyses revealed that the improvements in HbA1c with GLP-1RA treatment were associated with stroke prevention in DM patients with transient ischemic attack or stroke (RR, 0.840; 95% CI, 0.759–0.936; *p* = 0.001). Interestingly, the beneficial impact of improved glycemic control on stroke was unique to GLP-1RA and was not observed in SGLT-2i or insulin treatment [51].

It is worth mentioning that the maintenance of sinus rhythm is an important factor linked to a lower long-term risk of stroke and mortality. A meta-analysis of three propensity score-matched studies demonstrated that the use of GLP-1RA was associated with a lower risk of AF recurrence in patients receiving ablation therapy compared to controls (HR, 0.549; 95% CI, 0.315–0.956; *p* = 0.034) during a 12-month follow-up period. However, further research is needed to investigate the long-term efficacy of GLP-1RA in maintaining ablation outcomes [52].

#### 3.3.5. Sodium-Glucose Co-Transporter 2 Inhibitors

In the EMPA-REG (Empagliflozin Cardiovascular Outcome Event Trial in Type 2 Diabetes Mellitus Patients—Removing Excess Glucose) study, a reduction in the risk of a CV event was observed. This reduction was approximately 12% in the empagliflozin group in comparison to the placebo group during a follow-up period of 3.1 years. On the other hand, empagliflozin administration was associated with an increase in the risk of the occurrence of ischemic stroke; however, this increase was not statistically significant [30]. In the CANVAS (Canagliflozin Cardiovascular Assessment Study) trial, a lower risk of CV events was observed in the canagliflozin group compared to the placebo group (HR, 0.86; 95% CI, 0.75–0.97; *p* < 0.001 for non-inferiority and *p* = 0.02 for superiority). On the contrary, treatment with canagliflozin presented no benefit for stroke prevention, even though the point estimate was <1 for the occurrence of fatal or non-fatal stroke compared to the placebo group [31]. Moreover, DECLARE-TIMI 58 (Dapagliflozin Effect on CardiovascuLAR Events—Thrombolysis in Myocardial Infarction) trial suggested that patients who were treated with dapagliflozin showed no significant decrease in stroke risk in comparison to those who were administered placebo [32]. Finally, the VERTIS CV (Evaluation of Ertugliflozin Efficacy and Safety Cardiovascular Outcomes Trial) evaluated the effects of ertugliflozin compared to placebo on CV outcomes in patients with type 2 DM and atherosclerotic cardiovascular disease (ASCVD). The hazard ratio for a secondary outcome of stroke was 1.06 (95% CI, 0.82–1.37), consistent with findings from other SGLT-2i cardiovascular outcomes RCTs [33].

In a more recent population-based observational study, it was shown that SGLT-2i, namely dapagliflozin, empagliflozin, and canagliflozin, was associated with a reduction in mortality owing to CV events in comparison to other glucose-lowering drugs. Nevertheless, no difference was reported regarding the non-fatal stroke risk between the group that followed treatment with SGLT-2i and those who received placebo [42].

The effect of SGLT-2i on the occurrence of AF and stroke was explored in a large meta-analysis that included 56 RCTs and 111,773 patients. The authors found that SGLT-2i significantly reduced the incidence of AF (RR, 0.87; 95% CI, 0.76–0.99; *p* = 0.03), especially when used as monotherapy and in patients with type 2 DM. Nevertheless, the risk of stroke was not decreased after treatment with SGLT2i (RR, 0.97; 95% CI, 0.89–1.07; *p* = 0.56), and this finding was consistent when administered as monotherapy or combined therapy [53].

Table 1 presents the risk of stroke in major CV outcomes of antidiabetic drugs in clinical trials.

#### 3.3.6. Insulin

Insulin mainly exerts anti-atherosclerotic effects on vascular tissues. However, in subjects with insulin resistance, insulin administration can also induce pro-atherosclerotic effects, such as increased macrophage activation. It has also been suggested that elevated insulin levels induce pro-inflammatory responses, which may increase the risk of stroke. Experimental data, however, have shown that insulin reduces cerebral damage and neurological deficits due to ischemia or hypoperfusion [54]. There are only a few prospective intervention studies that have examined the CV effects of insulin therapy in type 2 DM. The UKPDS study revealed a statistically insignificant association of basal insulin with stroke risk [55]. Similarly, in the ORIGIN (Outcome Reduction with Initial Glargine Intervention) trial, no difference in stroke incidence was found between the group that received insulin glargine and the standard-care group (HR, 1.03; 95% CI, 0.89–1.21, *p* = 0.69) [34]. A recent meta-analysis showed that patients who received insulin had a lower risk of stroke compared to those under sulphonylureas [56]. Intriguingly, preliminary data from ongoing clinical trials suggest that once-weekly insulin treatments may be a promising approach to reduce the burden associated with DM and its complications [57].

#### 3.3.7. Combination of Antidiabetic Drugs

Combination therapy with GLP-1RA and SGLT-2i appears to have a beneficial impact on the incidence of CV events. It has been shown that the dual therapy results in a remarkable amelioration of endothelial, arterial, and cardiac function in conjunction with a greater improvement of oxidant and antioxidant biomarkers compared to each separate agent [10,11]. In a population-based cohort study, the use of combined GLP-1RA and SGLT-2i was associated with a lower risk of MACE in comparison with either agent class alone, without, however, a significant reduction in the risk of stroke (HR, 0.90; 95% CI, 0.48–1.67) [58]. Nonetheless, a recent meta-analysis that included real-world data from clinical practice in type 2 DM patients reported that in primary prevention, the use of a combination treatment with GLP-1RA and SGLT-2i was correlated with a 30% lower risk of major adverse cardiac and cerebrovascular events compared to other combination regimens (odds ratio (OR), 0.70; 95% CI, 0.50–0.98) [59].

According to a meta-analysis, treatment with a combination of SLGT-2i with pioglitazone improves glycemic control and reduces blood pressure and body weight in diabetic patients in comparison to treatments where pioglitazone is solely used. In addition, this combination may reduce the risk for CV death and renal disease, as well as AF. The authors conclude that even though an SGLT-2i is not directly correlated with a reduced risk of ischemic stroke, it has been associated with the mitigation of side effects, which are derived from the treatment with pioglitazone, such as fluid retention [60]. Therefore, SGLT-2i appears to be an optimal drug to include in antidiabetic treatment in combination with metformin and pioglitazone in type 2 DM patients with high CV risk [61].

Additionally, in a population-based case control study, patients who received combined therapy with insulin and metformin had a similar risk of stroke (OR, 0.98; 95% CI, 0.63–1.52) to those who treated with metformin in combination with a sulphonylurea. Moreover, meta-analysis with another observational study improved the risk estimate precision (RR, 0.92; 95% CI, 0.69–1.24) [43].

#### 3.3.8. Dual Glucose-Dependent Insulinotropic Polypeptide/Glucagon-Like Peptide-1 Receptor Agonists Agents

Tirzepatide is a first-in-class, dual glucose-dependent insulinotropic polypeptide (GIP)/GLP-1RA agent approved by the Food and Drug Administration (FDA), with initial data from phase 2 and 3 RCTs supporting its strong cardioprotective effects. A recent meta-analysis of 13 RCTs revealed that treatment with GLP-1RA or GIP/GLP-1RA was correlated with significant decrease in MACE (odds ratio, 0.87; 95% CI, 0.81–0.94; *p* < 0.01) [62]. Furthermore, subgroup analysis on GLP-1RA versus GIP/GLP-1RA showed no significant differences regarding their effects on MACE. Notably, four placebo-controlled RCTs on tirzepatide in patients with type 2 DM reported no stroke events [35,36,37,38]. Therefore, no inferences can be drawn regarding possible associations between tirzepatide and stroke occurrence based on the currently available data. Future large-scale RCTs are needed to elucidate the effects of GIP/GLP-1RA on primary and secondary stroke prevention.

## 4. Discussion

The findings of the present systematic review demonstrate that pioglitazone is the only antidiabetic treatment that significantly reduces the risk of stroke recurrence in patients with DM and an established stroke. In relation to primary prevention, the beneficial contribution of GLP-1RA seems to be important. In particular, semaglutide in the SUSTAIN-6 study reduced the risk of ischemic stroke by 39%, while dulaglutide in the REWIND study also showed a significant reduction of 26% in the incidence of non-fatal stroke. Furthermore, in a meta-analysis of RCTs and observational studies, metformin appeared to reduce the risk of stroke in the context of primary prevention, but its favorable effect in stroke prevention was not observed in patients who received combined treatment with metformin and other antihyperglycemic agents. Finally, DPP-4i showed CV safety regarding the risk of stroke, while SGLT-2i demonstrated safety and stroke risk neutrality. Similarly, insulin administration did not seem to affect the incidence of stroke.

The current clinical practice guidelines and position statements recommend the use of the newer antidiabetic drugs with a proven CV benefit, namely GLP-1RA and SGLT-2i, in patients with type 2 DM and ASCVD, including ischemic stroke. The American Diabetes Association suggests that in adults with type 2 DM and an established or high risk of ASCVD, heart failure, and/or chronic kidney disease, the treatment plan should include agents that reduce CV and kidney disease risk, such as GLP-1RA and/or SGLT-2i, for glycemic management and comprehensive CV risk reduction, independently of the value of baseline HbA1c and in consideration of person-specific factors and comorbidities [63]. The joint American Heart Association and American Stroke Association provided guidelines for patients with DM and transient ischemic attacks (TIA) or stroke, supporting the individualized use of glucose-lowering agents that are effective in the prevention of MACE [7]. Moreover, the American Association of Clinical Endocrinology recommends the use of GLP-1RA (semaglutide or dulaglutide) or pioglitazone independent of glycemic control or other antihyperglycemic medications in patients with type 2 DM and stroke or TIA [64]. The joint European Society of Cardiology and European Association for the Study of Diabetes recommends that the choice of antidiabetic therapy in patients with type 2 DM should be prioritized based on the presence of ASCVD or high/very high CV risk [65]. Of note, the European Stroke Organization (ESO) regarding the pharmacological interventions for long-term secondary prevention after TIA or ischemic stroke, suggests that in patients with previous TIA or ischemic stroke, who have insulin resistance or type 2 DM, pioglitazone should only be used to reduce the risk of recurrent stroke after careful consideration of the risk of side effects, such as heart failure, fracture, and bladder cancer, and counselling of the subject [66].

Preliminary evidence from phase 1 and 2 RCTs supports the potential cardioprotective effects of emerging agents for the treatment of type 2 DM and obesity. Retatrutide (LY3437943) is a triple agonist of the GIP, GLP-1, and glucagon receptors. A phase 2, double-blind, placebo-controlled RCT demonstrated that retatrutide administration in obese adults for 48 weeks resulted in a significant decrease in body weight and improvement of cardiometabolic parameters, including blood pressure and lipids [67]. Moreover, once-daily oral therapy with orforglipron, a nonpeptide GLP-1RA, was associated with weight reduction after 36 weeks of administration in adults with obesity [68]. On the other hand, preliminary trial results from co-administered once-weekly cagrilintide, a dual amylin and calcitonin receptor agonist, with once-weekly GLP-1RA semaglutide in subjects with type 2 DM, led to greater weight loss compared to either agent alone [69]. The dual agonists of GIP/GLP-1 maridebart cafraglutide (AMG 133) [70] and survodutide (BI 456906) [71] are investigational drugs with robust anti-obesity efficacy. Interestingly, the novel cannabinoid receptor-1 (CB1R) inverse agonist, INV-202/monlunabant in subjects with metabolic syndrome causes a remarkable reduction in body weight and improvement in the cardiometabolic profile without adverse effects [72]. In the future, further research is needed to shed more light on the potential CV and cerebrovascular effects of these new drugs.

The present systematic review has some limitations. First, in current clinical practice, the use of pioglitazone has been limited owing to concerns around side effects such as heart failure, weight gain, edema, and bone fractures. Second, the vast majority of the studies included in our review were RCTs. These trials have been used by international organizations and societies to plan clinical therapeutic decisions. However, RCTs often do not include sufficiently diverse populations to ensure broad generalizability of their results. Consequently, more real-world studies are required to explore the association between antidiabetic treatments and the prevention of ischemic stroke in the future.

## 5. Conclusions

Patients with DM have an increased risk for CV events, including ischemic stroke. Despite the fact that aggressive management of hyperglycemia has not been shown to decrease the risk of developing ischemic stroke, the effective management of hypertension appears to significantly reduce the corresponding risk. Regarding antidiabetic drugs, the findings of the present systematic review show that pioglitazone decreases the risk of recurrent stroke, whereas the improvement in glycemic control after treatment with GLP-1RA semaglutide and dulaglutide is associated with a reduction in the risk of ischemic stroke in diabetic subjects. Metformin monotherapy may reduce stroke risk, while DPP-4i, SGLT-2i, and insulin do not seem to affect the incidence of stroke. Further real-world studies are needed to provide additional data regarding the preventive effects of novel antidiabetic drugs, such as GIP/GLP-1RA, on stroke.

## Figures and Tables

**Table 1:** Stroke risk in major cardiovascular outcome trials of antidiabetic drugs.

Trial	Number of Participants	Drug	Class	Median Follow-Up Period (Years)	Stroke Risk	
					HR (95% CI)	*p* Value
SAVOR-TIMI 53 [17]	16,492	Saxagliptin	DPP-4i	2.1	1.11 (0.88–1.39)	0.38
EXAMINE [18]	5380	Alogliptin	DPP-4i	1.5	0.91 (0.55–1.50)	0.71
TECOS [19]	14,671	Sitagliptin	DPP-4i	3.0	0.97 (0.79–1.19)	0.76
CARMELINA [20]	6979	Linagliptin	DPP-4i	2.2	0.91 (0.67–1.23)	0.53
CAROLINA [21]	6979	Linagliptin	DPP-4i	6.3	0.86 (0.66–1.12)	0.27
ELIXA [22]	6068	Lixisenatide	GLP-1RA	2.1	1.12 (0.79–1.58)	0.54
LEADER [23]	9340	Liraglutide	GLP-1RA	3.8	0.86 (0.71–1.06)	0.16
SUSTAIN-6 [24]	3297	Semaglutide	GLP-1RA	2.1	0.61 (0.38–0.99)	0.04
SELECT * [25]	17,604	Semaglutide	GLP-1RA	3.3	0.93 (0.74–1.15)	NR
EXSCEL [26]	14,752	Exenatide	GLP-1RA	3.2	0.85 (0.70–1.03)	0.095
REWIND [27]	9901	Dulaglutide	GLP-1RA	5.4	0.76 (0.61–0.95)	0.017
PIONEER-6 [29]	3183	Semaglutide	GLP-1RA	1.3	0.74 (0.35–1.57)	NR
EMPA-REG [30]	7020	Empagliflozin	SGLT-2i	3.1	1.18 (0.89–1.56)	0.26
CANVAS [31]	10,142	Canagliflozin	SGLT-2i	3.6	0.87 (0.69–1.09)	0.23
DECLARE-TIMI 58 [32]	17,160	Dapagliflozin	SGLT-2i	4.2	1.01 (0.84–1.21)	0.53
VERTIS CV [33]	8246	Ertugliflozin	SGLT-2i	3.5	1.06 (0.82–1.37)	NR

HR, hazard ratio; CI, confidence interval; DPP-4i, dipeptidyl peptidase 4 inhibitors; GLP-1RA, glucagon-like peptide-1 receptor agonists; SGLT-2i, sodium-glucose co-transporter 2 inhibitors; NR, not reported. * This trial included overweight or obese patients without diabetes.

## Data Availability

Data are available from the corresponding author upon reasonable request.
